# Substantial declines in eGFR after initiation of SGLT2-inhibitors associate with increased short-term mortality in hospitalized patients with CKD

**DOI:** 10.1093/ckj/sfag386

**Published:** 2026-10-29

**Authors:** Qi Gao, Zhou Ma, Weirong Ren, Yaping Yu, Lunlun Wu, Zhiqin Zhang, Zhongcai Wu, Shiyu Zhou, Licong Su, Yanqin Li, Fan Luo, Yuxin Lin, Junwu Dong, Honglan Wei

**Affiliations:** Department of Nephrology, Wuhan Fourth Hospital, Wuhan Clinical Research Center for Metabolic Chronic Kidney Disease, Wuhan, Hubei, China; Department of Nephrology, Wuhan Fourth Hospital, Wuhan Clinical Research Center for Metabolic Chronic Kidney Disease, Wuhan, Hubei, China; Department of Nephrology, Wuhan Fourth Hospital, Wuhan Clinical Research Center for Metabolic Chronic Kidney Disease, Wuhan, Hubei, China; Department of Nephrology, Wuhan Fourth Hospital, Wuhan Clinical Research Center for Metabolic Chronic Kidney Disease, Wuhan, Hubei, China; School of Medicine, Jianghan University, Wuhan, Hubei, China; Department of Nephrology, Wuhan Fourth Hospital, Wuhan Clinical Research Center for Metabolic Chronic Kidney Disease, Wuhan, Hubei, China; Department of Nephrology, Wuhan Fourth Hospital, Wuhan Clinical Research Center for Metabolic Chronic Kidney Disease, Wuhan, Hubei, China; Division of Nephrology, National Clinical Research Center for Kidney Disease, State Key Laboratory of Organ Failure Research, Nanfang Hospital, Southern Medical University, Guangzhou, Guangdong, China; Division of Nephrology, National Clinical Research Center for Kidney Disease, State Key Laboratory of Organ Failure Research, Nanfang Hospital, Southern Medical University, Guangzhou, Guangdong, China; Division of Nephrology, National Clinical Research Center for Kidney Disease, State Key Laboratory of Organ Failure Research, Nanfang Hospital, Southern Medical University, Guangzhou, Guangdong, China; Division of Nephrology, National Clinical Research Center for Kidney Disease, State Key Laboratory of Organ Failure Research, Nanfang Hospital, Southern Medical University, Guangzhou, Guangdong, China; Division of Nephrology, National Clinical Research Center for Kidney Disease, State Key Laboratory of Organ Failure Research, Nanfang Hospital, Southern Medical University, Guangzhou, Guangdong, China; Department of Nephrology, Wuhan Fourth Hospital, Wuhan Clinical Research Center for Metabolic Chronic Kidney Disease, Wuhan, Hubei, China; Department of Nephrology, Wuhan Fourth Hospital, Wuhan Clinical Research Center for Metabolic Chronic Kidney Disease, Wuhan, Hubei, China

**Keywords:** China Renal Data System, chronic kidney disease, estimated glomerular filtration rate, mortality, sodium–glucose cotransporter 2 inhibitors

## Abstract

**Background:**

Sodium–glucose cotransporter 2 inhibitors (SGLT2i) are recommended as first-line therapy for patients with chronic kidney disease (CKD). However, the prevalence and prognostic significance of a large early estimated glomerular filtration rate (eGFR) dip after SGLT2i initiation in real-world CKD populations remain unclear.

**Methods:**

We conducted a multicenter retrospective cohort study using the China Renal Data System. Adult hospitalized patients with eGFR ≥20 ml/min/1.73 m^2^ who newly initiated SGLT2i were included. Exposure was defined as an eGFR decline ≥20% within 14–30 days after treatment initiation. Follow-up began 30 days after initiation. The outcomes were all-cause mortality within 45 days and all-cause mortality from 46 days to 1 year. Inverse probability of treatment weighting-weighted landmark Cox model was performed to evaluate these associations.

**Results:**

Among 1258 eligible patients (mean age 64.4 years; 69.3% male), 22.5% experienced an early eGFR dip ≥20%. Higher incidence rates were observed in older patients, those with heart failure, diuretic use, and CKD stages 2–4. Compared with an eGFR dip <20%, an eGFR dip ≥20% was associated with an increased risk of all-cause mortality within 45 days (adjusted hazard ratio [aHR], 8.08; 95% confidence interval [CI], 3.41–19.13). However, no significant association was observed between an eGFR dip ≥20% and all-cause mortality from 46 days to 1 year (aHR, 0.91; 95% CI, 0.48–1.74).

**Conclusions:**

A substantial eGFR decline after SGLT2i initiation was not uncommon and was associated with increased short-term all-cause mortality, highlighting the importance of close kidney function monitoring and individualized clinical assessment of treatment continuation.

## Graphical Abstract

**Graphical Abstract:**
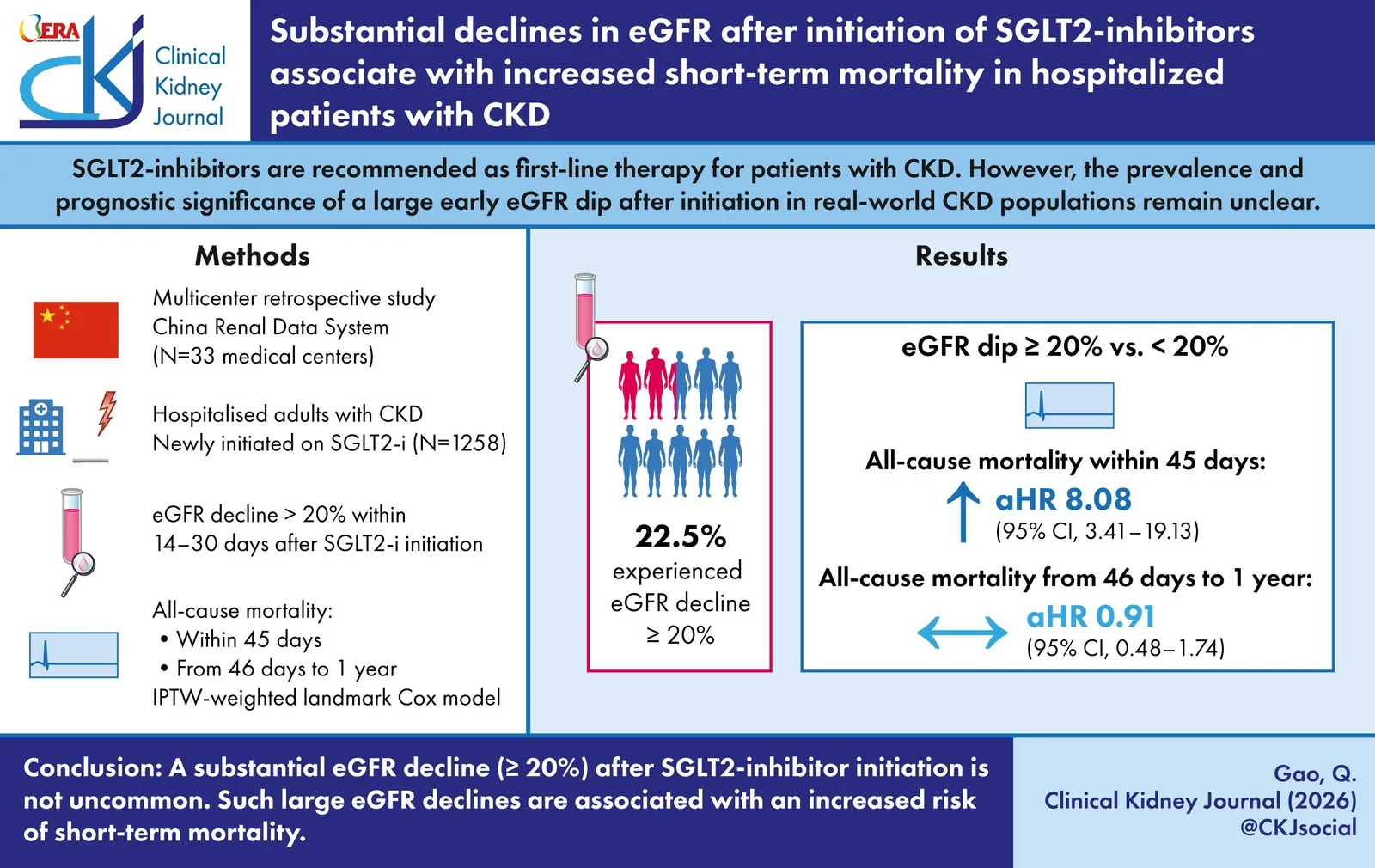

KEY LEARNING POINTS **What was known:**

- Sodium–glucose cotransporter 2 inhibitors are widely used in patients with chronic kidney disease, but some experience an early decline in kidney function after initiation. While small declines are expected, the clinical significance of larger drops is uncertain.

**This study adds:**

- In this multicenter study, nearly one-quarter of patients experienced an estimated glomerular filtration rate (eGFR) dip ≥20%, which was associated with an eight-fold higher risk of short-term mortality.

**Potential impact:**

- Our study highlights the importance of close monitoring in high-risk patients with large declines in eGFR.

## INTRODUCTION

According to the 2023 Global Burden of Disease (GBD) study, nearly 800 million people worldwide are affected by chronic kidney disease (CKD), which is projected to become the fifth leading cause of death globally by 2040 [1, 2]. China currently has the largest population of patients with CKD, with a prevalence of ~8.2% [3]. CKD is a chronic and progressive disease, and once patients progress to end-stage kidney disease (ESKD), long-term kidney replacement therapies are required, placing a substantial burden on patients, families, and healthcare systems.

Sodium–glucose cotransporter 2 inhibitors (SGLT2i) are a novel class of oral glucose-lowering agents. In recent years, accumulating evidence has demonstrated that SGLT2i confer renoprotective effects in patients with CKD, both with and without diabetes mellitus, that are independent of their glucose-lowering properties. SGLT2i inhibit tubular reabsorption of glucose and urinary sodium, thereby reducing glomerular hyperfiltration [4, 5]. In addition, SGLT2i exert anti-inflammatory and antifibrotic effects through multiple mechanisms, including enhanced ketone body production, reduced macrophage infiltration into renal tissue, and downregulation of pro-inflammatory cytokine expression, collectively slowing the progression of tubulointerstitial fibrosis [6–8]. Consequently, SGLT2i have now been established as first-line therapy for the management of CKD [9].

Several randomized controlled trials (RCTs) have reported that the reduction in glomerular hyperfiltration following SGLT2i initiation may lead to a transient decline in estimated glomerular filtration rate (eGFR) within 2–4 weeks, a phenomenon commonly referred to as the “initial dip” [10, 11]. In some patients, this decline may exceed 10% from baseline, followed by a gradual recovery and stabilization of kidney function [12–14]. Moreover, *post hoc* analyses of multiple RCTs have suggested that a modest eGFR reduction (with a threshold of 10%) does not significantly increase renal or cardiovascular risk and may even be associated with improved long-term renal outcomes [14, 15].

However, patients in real-world clinical settings are more heterogeneous and frequently present with multiple comorbidities. The proportion of individuals who experience a rapid and substantial decline in kidney function (eGFR dip ≥20%) after initiating SGLT2i, as well as the clinical implications of such declines, remain unclear. This uncertainty may deter clinicians from continuing SGLT2i therapy in practice. To address this knowledge gap, we aimed to investigate the association between an eGFR dip ≥20% and all-cause mortality using a large multicenter database.

## MATERIALS AND METHODS

### Study design, data source, and population

Data for this multicenter retrospective real-world study were obtained from the China Renal Data System (CRDS), a nationwide, population-based database jointly established by the National Clinical Research Center for Kidney Disease and the Chinese Center for Disease Control and Prevention (CDC). The CRDS comprises electronic health records from >27 million individuals covering 33 urban academic hospitals across China. Patient-level data from each participating center were systematically cleaned, standardized, anonymized, and centrally integrated at the CRDS data center located in Guangzhou. The database contains comprehensive inpatient and outpatient information, including demographic characteristics, admission and discharge diagnoses, laboratory measurements, prescriptions, surgical procedures, vital signs, and other clinical records [16–19]. Mortality data were obtained through linkage with the Cause of Death Reporting System of the China CDC. The CRDS has received ISO9001 quality certification and approval from the China Office of Human Genetic Resources for Data Preservation Application (approval number: 2021-BC0037). The study protocol was approved by the Medical Ethics Committee of Nanfang Hospital, Southern Medical University (approval numbers: NFEC201905-K10 and NFEC-2023-409) and Ethics Committee of Wuhan Fourth Hospital (approval number: KY2024-199-001). The requirement for informed consent was waived owing to the use of anonymized data. This study was conducted in accordance with the Declaration of Helsinki and according to the Strengthening the Reporting of Observational Studies in Epidemiology (STROBE) guidelines [20].

We initially identified adult hospitalized patients aged 18–100 years with CKD who initiated SGLT2i therapy (Fig. S1). CKD was defined by the presence of at least one of the following criteria [19]: (1) an eGFR <60 ml/min/1.73 m^2^ persisting for >90 days; (2) persistent proteinuria for >90 days; or (3) International Classification of Diseases, 10th Revision (ICD-10) codes, including N02-N08, N11, N15.0, N18-N19, N25-N27, N28.818, N39.1, I12-I13, I15.102-I15.103, E10.2, E11.2, E12.2, E13.2, and E14.2. The index date was defined as the date of the first SGLT2i prescription during the patient’s hospitalization recorded in the CRDS database. The exclusion criteria were as follows: (1) Less than 1 year of prescription records prior to the index date; (2) previous use of SGLT2i before the index date; (3) absence of serum creatinine (SCr) measurements within 30 days before the index date; (4) suspected acute kidney injury (AKI) at the index date, defined as a >1.5-fold fluctuation in SCr within 30 days prior to the index date; (5) eGFR <20 ml/min/1.73 m^2^, prior maintenance dialysis, or kidney transplant before the index date; (6) pregnancy at the index date; (7) absence of urine albumin-to-creatinine ratio (UACR) measurements within 365 days before the index date; (8) lack of repeated SCr measurements within 14–30 days after the index date; and (9) death or censoring before the enrollment date. A total of 1258 CKD patients who initiated SGLT2i therapy for the first time were included in the final analysis.

### Exposure and outcomes

The eGFR at the index date was defined as the most recent value within 30 days prior to the index date. eGFR was calculated using the ethnicity-independent CKD Epidemiology Collaboration equation [21]. Exposure was defined as a decline in eGFR of >20% occurring within 14–30 days after initiation of SGLT2i therapy, whereas the unexposed group was defined as having a decline in eGFR of <20% during the same period. For patients with multiple eGFR measurements within 14–30 days after SGLT2i initiation, the lowest eGFR value was used. The enrollment date was defined as 30 days after the index date for both the exposed and unexposed groups, with day 30 designated as the landmark time point (Fig. S1). The outcomes were all-cause mortality within 45 days and all-cause mortality from 46 days to 1 year. Patients were followed from the enrollment date until death or the last available follow-up date recorded in the database, whichever occurred first.

### Covariates

We collected patients’ demographic information (age and sex), vital signs (systolic blood pressure [SBP] and diastolic blood pressure [DBP]), comorbidities (identified using the ICD-10 codes within 365 days prior to the index date), medication use (defined as any prescription within 365 days prior to the index date), and baseline laboratory measurements (defined as the most recent values within 365 days prior to the index date). The timing of covariates assessment is illustrated in Fig. S1. The list of medications and diagnoses with corresponding anatomical therapeutic chemical classification system and ICD-10 codes was shown in Table S1.

### Handling of missing values

The proportion of missing data for all covariates was <20% (Table S2). Missing values were assumed to be missing at random and were imputed using a random forest-based algorithm implemented in the missRanger package in R.

### Statistical analysis

Baseline characteristics were summarized as means with standard deviations, medians with interquartile ranges (25^th^, 75^th^ percentiles), and counts with percentages. Between-group comparisons were performed using analysis of variance (ANOVA), the Wilcoxon rank-sum test, the chi-square test, or Fisher’s exact test, as appropriate.

Incidence rates of an eGFR dip ≥20%, expressed per 100 person-months with corresponding 95% confidence intervals (CIs), were calculated for different subgroups. Incidence rates were compared using Poisson regression models with person-month included as an offset. Inverse probability of treatment weighting (IPTW)-weighted landmark Kaplan–Meier (KM) curves were constructed to illustrate cumulative all-cause mortality, with the cutoff for the two follow-up periods set at 45 days after the landmark time point, describing mortality within the first 45 days and from day 46 to 1 year after the landmark. IPTW-weighted landmark Cox proportional hazards (PHs) models were used to evaluate the association between an eGFR dip ≥20% and mortality, with results reported as hazard ratios (HRs) and corresponding 95% CIs. The R function cox.zph() was used to test whether the PH assumption was violated. In Model 1, analyses were adjusted for sex, age, and the Charlson comorbidity index (CCI). Model 2 was further adjusted for UACR, use of angiotensin-converting enzyme inhibitors or angiotensin receptor blockers (ACEI/ARBs), and diuretics. To account for the different exposure windows, propensity scores (PSs) for experiencing an eGFR dip ≥20% were estimated separately for the 45 days and 46 days to 1 year windows using multivariable logistic regression models that included all covariates in Model 2 (Fig. S2). For IPTW, weights were defined as 1/PS for patients with an eGFR dip ≥20% and 1/(1-PS) for those with an eGFR dip <20%. Meanwhile, we applied weight trimming by restricting the weights to the 2.5^th^ and 97.5^th^ percentiles to reduce the influence of extreme weights. Meanwhile, restricted cubic splines (RCS) were used to characterize the dose–response relationship between the percentage of eGFR decline and the risk of all-cause mortality within 45 days and from 46 days to 1 year. We further evaluated eGFR decline as a continuous variable (per 5% decrease in eGFR) using IPTW-weighted Cox models to further examine the association between eGFR decline and mortality.

### Subgroup analyses

We explored potential effect modification in the association between eGFR dip and all-cause mortality across several predefined subgroups. Patients were stratified by age (≤60 vs. >60 years), sex (female vs. male), CKD stages (1–2 vs. 3–4), UACR (<300 vs. ≥300 mg/g), SGLT2i subtypes (dapagliflozin vs. non-dapagliflozin), hypertension (no vs. yes), diabetes mellitus (no vs. yes), heart failure (no vs. yes), and use of ACEI/ARB (no vs. yes).

### Sensitivity analyses

To assess the robustness of our findings, we conducted several sensitivity analyses. First, given the substantially increased mortality risk among patients with malignancy, we repeated the analyses after excluding 148 patients with malignant tumors at baseline. Sensitivity analysis 1 was also conducted using an IPTW-weighted Cox model. Second, we replaced IPTW with overlap weighting (OW), another commonly used PS-based weighting approach [22], and repeated the analyses. For OW, weights were defined as 1-PS for patients with an eGFR dip ≥20% and PS for those with an eGFR dip <20%. Finally, we conducted *E*-value analyses to assess the robustness of the observed associations to potential unmeasured confounding [23].

### Additional analyses

We evaluated alternative exposure definitions using different thresholds (≥10% and ≥30% eGFR decline) and applied IPTW-adjusted Cox PHs models to examine the association between eGFR decline and mortality.

All statistical analyses were performed using R software (version 4.5.1; http://www.r-project.org/). All statistical tests were two-sided and a *P*-value < .05 was considered statistically significant.

## RESULTS

### Baseline characteristics of study population

A total of 1258 CKD patients who newly initiated SGLT2i were included in this study. Baseline characteristics of the study population were summarized in Table 1. The mean age of the cohort was 64.4 ± 14.5 years, and 69.3% of the participants were male. The median baseline UACR was 75.5 [15.3, 596.6] mg/g. More than half of the patients received dapagliflozin as their SGLT2i subtype. The median eGFR prior to SGLT2i initiation was 66.8 [49.8, 89.9] ml/min/1.73 m², with the majority of patients classified as CKD stages 2–3. After 14–30 days of SGLT2i therapy, the median eGFR was 63.5 [43.7, 86.2] ml/min/1.73 m². 73.8% and 32.3% of patients had diabetes mellitus and heart failure, respectively, and 68.0% were concurrently treated with ACEI/ARB. Overall, ~22.5% of patients experienced a short-term eGFR dip of >20% following their initiation of SGLT2i therapy. Compared with those with eGFR dip <20%, patients with eGFR dip ≥20% were older (68.3 vs. 63.3 years), had a lower proportion of males (64.7% vs. 70.7%), lower baseline eGFR (60.4 vs. 69.0 ml/min/1.73 m²), higher prevalence of heart failure (47.3% vs. 27.9%), and were more likely to use diuretics (67.1% vs. 44.7%). No significant differences were observed between groups in the prevalence of hypertension or ACEI/ARB use. Among patients with an eGFR dip ≥20%, eGFR declined from 60.4 [47.6, 79.6] ml/min/1.73 m² at index date to 39.7 [26.4, 52.3] ml/min/1.73 m² within 2–4 weeks after initiation of SGLT2i therapy. Baseline characteristics stratified by all-cause mortality were presented in Table S3.

**Table 1:** Baseline characteristics of hospitalized patients with CKD stratified by initial dip in eGFR.

Variable		Initial dip in eGFR		
	Overall (*n* = 1258)	<20% (*n* = 975)	≥20% (*n* = 283)	*P* value
Age, year	64.4 (14.5)	63.3 (14.6)	68.3 (13.6)	<.001
Male, *n* (%)	872 (69.3)	689 (70.7)	183 (64.7)	.064
SCr at index date, mmol/l	99.0 [76.0, 127.0]	98.0 [73.0, 125.9]	102.0 [84.8, 130.5]	.007
eGFR at index date, ml/min/1.73 m^2^	66.8 [49.9, 89.9]	69.0 [51.2, 93.1]	60.4 [47.6, 79.6]	<.001
CKD stages, *n* (%)				<.001
1	313 (24.9)	270 (27.7)	43 (15.2)	
2	430 (34.2)	329 (33.7)	101 (35.7)	
3	446 (35.5)	324 (33.2)	122 (43.1)	
4	69 (5.5)	52 (5.3)	17 (6.0)	
eGFR at enrollment date, ml/min/1.73 m^2^	63.5 [43.7, 86.2]	70.7 [53.0, 92.9]	39.7 [26.4, 52.3]	<.001
UACR, mg/g	75.5 [15.3, 596.6]	75.5 [15.3, 631.2]	60.5 [14.1, 363.9]	.075
SGLT2i subtypes, *n* (%)				.231
Dapagliflozin	841 (66.9)	650 (66.7)	191 (67.5)	
Canagliflozin	175 (13.9)	142 (14.6)	33 (11.7)	
Empagliflozin	235 (18.7)	176 (18.1)	59 (20.8)	
Ertugliflozin	7 (0.6)	7 (0.7)	0 (0.0)	
SBP, mmHg	131.4 (21.3)	131.0 (20.6)	132.9 (23.6)	.196
DBP, mmHg	77.1 (13.4)	77.4 (13.2)	75.8 (14.1)	.063
CCI	8.0 [6.0, 10.0]	8.0 [5.0, 10.0]	8.0 [6.0, 10.0]	.048
Diabetes mellitus, *n* (%)	929 (73.8)	734 (75.3)	195 (68.9)	.038
Hypertension, *n* (%)	829 (65.9)	638 (65.4)	191 (67.5)	.568
Heart failure, *n* (%)	406 (32.3)	272 (27.9)	134 (47.3)	<.001
Acute coronary syndrome, *n* (%)	507 (40.3)	375 (38.5)	132 (46.6)	.016
Cerebrovascular disease, *n* (%)	439 (34.9)	350 (35.9)	89 (31.4)	.190
Stroke, *n* (%)	330 (26.2)	260 (26.7)	70 (24.7)	.566
COPD, *n* (%)	258 (20.5)	205 (21.0)	53 (18.7)	.448
Malignant tumor, *n* (%)	148 (11.8)	124 (12.7)	24 (8.5)	.065
ACEI/ARB, *n* (%)	856 (68.0)	651 (66.8)	205 (72.4)	.084
Beta block, *n* (%)	554 (44.0)	412 (42.3)	142 (50.2)	.022
Calcium channel blocker, *n* (%)	655 (52.1)	500 (51.3)	155 (54.8)	.334
Diuretic, *n* (%)	626 (49.8)	436 (44.7)	190 (67.1)	<.001
Insulin, *n* (%)	746 (59.3)	574 (58.9)	172 (60.8)	.613
Metformin, *n* (%)	443 (35.2)	365 (37.4)	78 (27.6)	.003
DPP4i, *n* (%)	256 (20.3)	201 (20.6)	55 (19.4)	.726
Sulfonylurea, *n* (%)	181 (14.4)	149 (15.3)	32 (11.3)	.114
Statin, *n* (%)	895 (71.1)	688 (70.6)	207 (73.1)	.442
NSAIDs, *n* (%)	659 (52.4)	505 (51.8)	154 (54.4)	.478
Proton pump inhibitor, *n* (%)	610 (48.5)	452 (46.4)	158 (55.8)	.006
Contrast, *n* (%)	285 (22.7)	221 (22.7)	64 (22.6)	1.000
Cardiac major surgery, *n* (%)	13 (1.0)	10 (1.0)	3 (1.1)	1.000
CABG, *n* (%)	60 (4.8)	44 (4.5)	16 (5.7)	.526
BUN, mmol/l	7.2 [5.5, 9.6]	7.0 [5.3, 9.4]	7.8 [6.2, 10.2]	<.001
Uric acid, mmol/l	380.0 [301.0, 475.0]	375.0 [296.5, 468.0]	399.0 [314.0, 499.5]	.004
Glucose, mmol/l	7.8 [5.7, 11.4]	7.8 [5.8, 11.5]	7.5 [5.7, 10.5]	.224
Triglycerides, mmol/l	1.5 [1.1, 2.4]	1.6 [1.1, 2.4]	1.5 [1.0, 2.3]	.179
Total cholesterol, mmol/l	4.4 [3.5, 5.5]	4.4 [3.5, 5.5]	4.3 [3.5, 5.4]	.646
LDL-C, mmol/l	2.6 [1.9, 3.3]	2.6 [1.9, 3.4]	2.6 [1.9, 3.2]	.671
HDL-C, mmol/l	1.0 [0.9, 1.3]	1.0 [0.9, 1.3]	1.0 [0.8, 1.2]	.025
Albumin, g/l	38.0 [33.3, 41.4]	38.4 [33.9, 41.7]	36.1 [31.7, 40.3]	<.001
Hemoglobin, g/l	128.0 [112.0, 143.0]	129.0 [114.0, 145.0]	122.0 [105.5, 138.0]	<.001
Sodium, mmol/l	139.6 [137.0, 141.7]	139.5 [137.1, 141.]	140.0 [136.7, 141.9]	.628
Potassium, mmol/l	4.1 [3.7, 4.4]	4.1 [3.7, 4.3]	4.0 [3.7, 4.4]	.582
D-dimer, μg/ml	0.5 [0.3, 1.1]	0.5 [0.3, 1.0]	0.7 [0.4, 1.5]	<.001

Data were presented as mean (standard deviation), median [25^th^, 75^th^ percentiles], or frequencies (percentages).

**Abbreviations**: DPP4i, dipeptidyl peptidase 4 inhibitors; NSAIDs, nonsteroidal anti-inflammatory drugs; CABG, coronary artery bypass grafting; BUN, blood urea nitrogen; LDL-C, low-density lipoprotein cholesterol; HDL-C, high-density lipoprotein cholesterol.

### Incidence rate of eGFR dip ≥20% in subgroups

The incidence rates of eGFR dip ≥20% across different subgroups were shown in Fig. 1. Higher incidence rates were observed in patients aged > 60 years (1.8 [1.5–2.0] vs. 1.0 [0.8–1.3] per 100 person-months, *P* < .001), patients with heart failure (2.4 [2.0–2.9] vs. 1.1 [0.9–1.3] per 100 person-months, *P* < .001), patients without diabetes mellitus (2.0 [1.6–2.5] vs. 1.3 [1.1–1.5] per 100 person-months, *P* < .001), and patients using diuretics (2.1 [1.8–2.4] vs. 0.9 [0.7–1.1] per 100 person-months, *P* < .001). Notably, compared with CKD stage 1 (0.9 [0.6–1.2] per 100 person-months), the incidence rates were significantly higher in CKD stage 2 (1.6 [1.3–1.9], *P* = .001), stage 3 (1.8 [1.5–2.3], *P* < .001), and CKD stage 4 (1.8 [1.0–2.8], *P* = .015). No significant differences were observed across subgroups defined by sex, UACR levels, presence of hypertension, chronic obstructive pulmonary disease (COPD), cerebrovascular disease, or use of ACEI/ARB (all *P* > .05).

**Figure 1:**
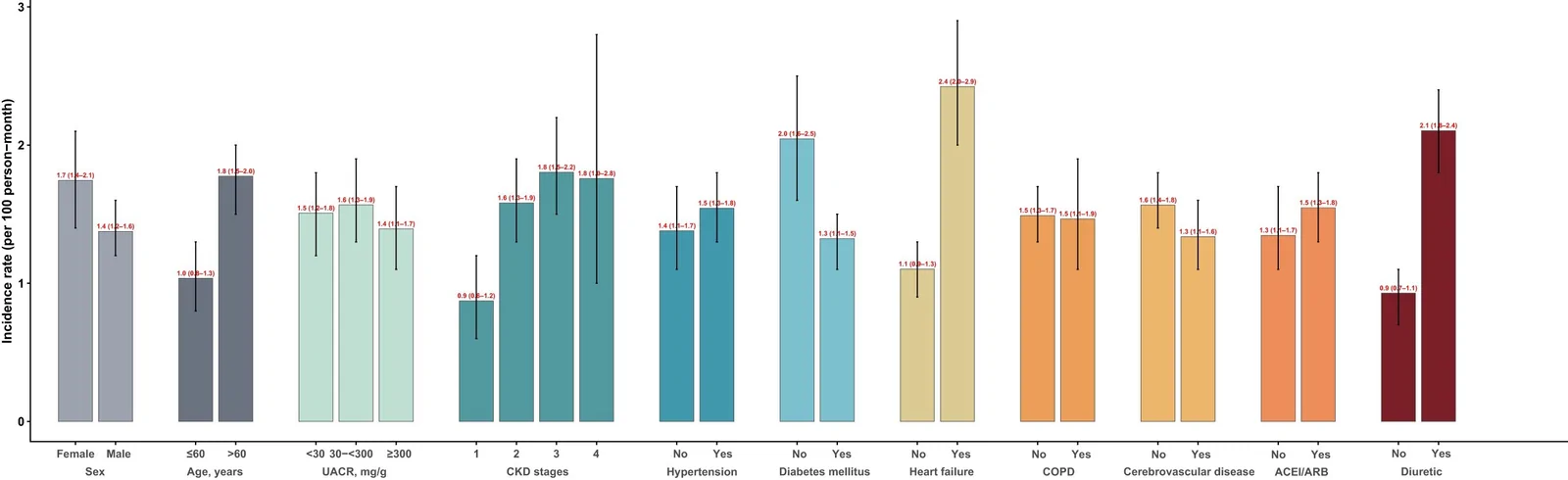
Incidence rates of early eGFR decline ≥20% across various subgroups. The incidence rates are presented as per 100 person-months. Error bars represent the 95% CIs.

### Association of eGFR dip with all-cause mortality

The median follow-up duration was 12.5 [5.7, 22.9] months. We selected a 1-year follow-up window and used day 45 as the piecewise time point. The IPTW-weighted landmark KM curves for the exposed and unexposed groups were shown in Fig. 2. In the multivariable Model 2, adjusted for sex, age, CCI, UACR, ACEI/ARB use, and diuretic use, no violation of the PH assumption was observed (*P* > .05; Table S4). Within the first 45 days, 20 patients (5.2 per 100 person-months) in the eGFR dip ≥20% group and 8 patients (0.6 per 100 person-months) in the eGFR dip <20% group experienced all-cause mortality (Table 2). In Model 2, an eGFR dip ≥20% was associated with a higher risk of all-cause mortality within 45 days (HR, 8.08, 95% CI, 3.41–19.13, Table 2). However, among patients who survived beyond 45 days, no significant association was observed between an eGFR dip ≥20% and subsequent all-cause mortality from day 46 to 1 year (HR, 0.91, 95% CI, 0.48–1.74, Table 2).

**Figure 2:**
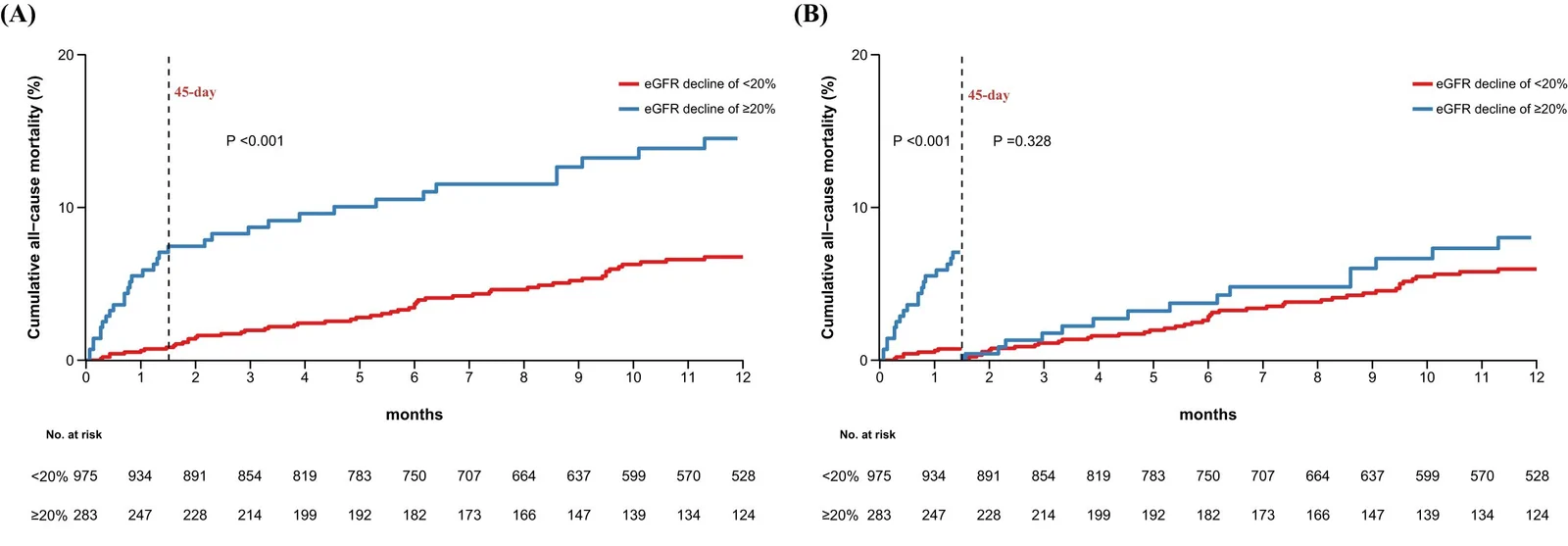
KM curves for cumulative all-cause mortality stratified by initial dip in eGFR. (A) Overall follow-up without piecewise analysis; (B) piecewise analysis using 45 days as the time point, showing all-cause mortality within 45 days and from 46 days to 1 year. *P* values were calculated using the weighted log-rank test.

**Table 2:** Association between early eGFR decline and risk of all-cause mortality using a 45-day piecewise analysis with IPTW-weighted landmark Cox models in hospitalized patients with CKD.

Initial dip in eGFR	Total, *N*	No. of events (per 100 person-months)	Crude model		Model 1		Model 2	
			HR [95% CI]	*P* value	HR [95% CI]	*P* value	HR [95% CI]	*P* value
**Using a ≥20% decline in eGFR as the exposure definition**								
**All-cause mortality within 45 days**								
<20%	975	8 (0.6)	Ref		Ref		Ref	
≥20%	283	20 (5.2)	8.79 [3.80, 20.32]	<.001	8.44 [3.59, 19.81]	<.001	8.08 [3.41, 19.13]	<.001
**All-cause mortality from 46 days to 1 year**								
<20%	915	46 (0.6)	Ref		Ref		Ref	
≥20%	235	14 (0.8)	1.07 [0.58, 2.00]	.824	1.01 [0.54, 1.91]	.970	0.91 [0.48, 1.74]	.778
**Per 5% decline**								
**All-cause mortality within 45 days**								
Per 5% decline	1258	28 (1.6)	1.24 [1.16, 1.32]	<.001	1.23 [1.15, 1.31]	<.001	1.22 [1.14, 1.31]	<.001
**All-cause mortality from 46 days to 1 year**								
Per 5% decline	1150	60 (0.6)	1.01 [0.96, 1.06]	.710	1.00 [0.95, 1.05]	.915	0.99 [0.94, 1.04]	.796

a A 45-day piecewise analysis was applied, with follow-up divided into 0–45 days and 46 days to 1 year.

**Abbreviations**: Ref, reference; EPV, events per variable.

**Model 1**: adjusted for sex, age, and CCI.

**Model 2**: Model 1 + further adjusted for UACR, ACEI/ARB, and diuretic.

In Model 2, the EPV values were 4 for all-cause mortality within 45 days and 8.6 for all-cause mortality from 46 days to 1 year.

We further examined the dose–response relationship between the percentage of eGFR decline and the risk of all-cause mortality within 45 days and from 46 days to 1 year (Fig. 3). We observed a linear association between eGFR decline and all-cause mortality within 45 days, whereas no significant association was found for mortality from 46 days to 1 year. Each 5% decrease in eGFR was associated with a 1.22-fold higher risk of all-cause mortality within 45 days (HR, 1.22; 95% CI, 1.14–1.31, Table 2).

**Figure 3:**
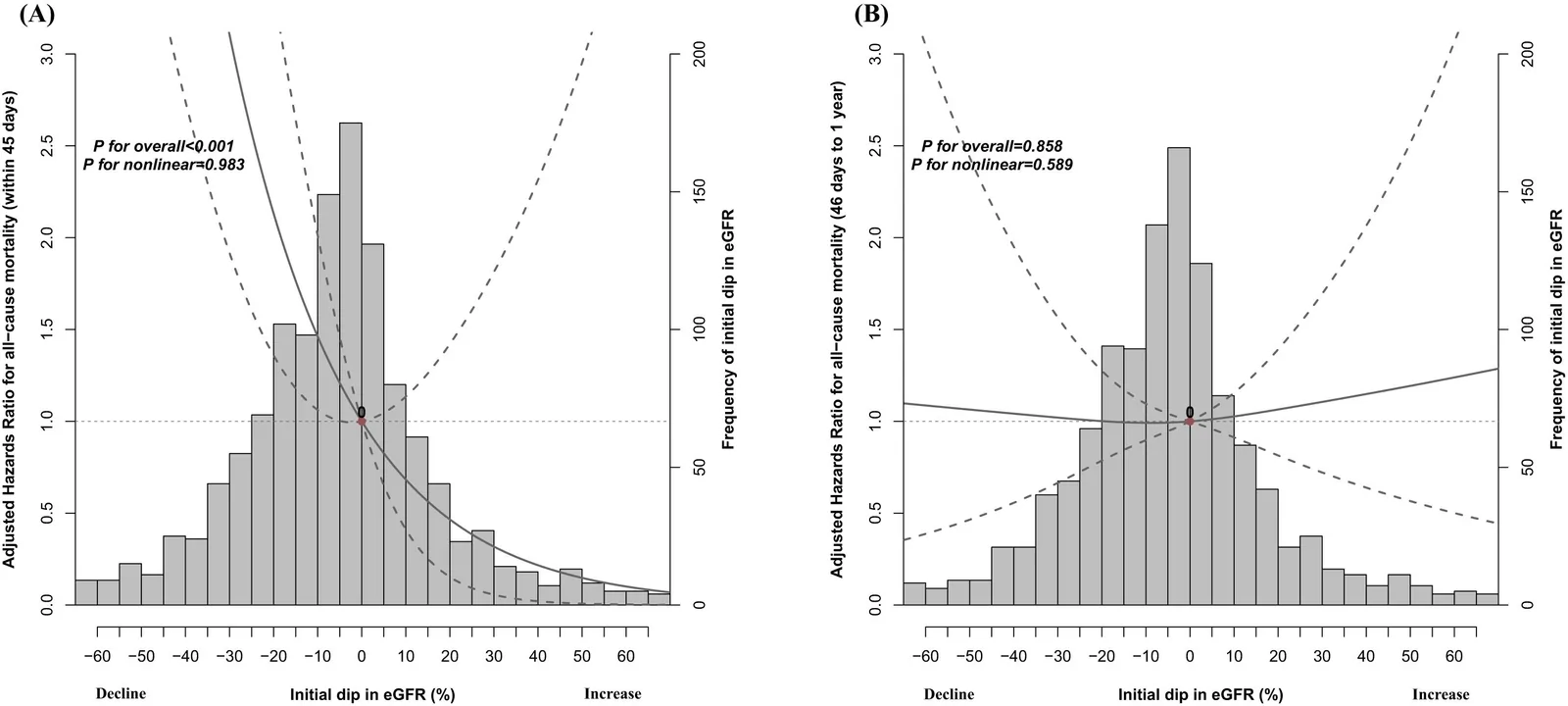
Relationship between early eGFR decline and risk of all-cause mortality based on RCS. **Abbreviation**: Ref, reference. Negative values indicate a decline in eGFR, whereas positive values indicate an increase in eGFR. The model was adjusted for sex, age, CCI, UACR, ACEI/ARB, and diuretic.

### Subgroup analyses

Subgroup analyses demonstrated consistent associations between an eGFR dip ≥20% and the risk of all-cause mortality (Table S5). No significant effect modification was observed by age, sex, CKD stage, UACR, SGLT2i subtype, diabetes mellitus, heart failure, or ACEI/ARB use, significantly modified the association between eGFR dip and all-cause mortality (all *P* for interaction >.05, Table S5). Notably, due to the limited number of events and small sample sizes in certain subgroups, HRs and *P* values for interaction could not be reliably estimated. Therefore, the results of the subgroup analyses should be interpreted with caution.

### Sensitivity analyses

Results from multiple sensitivity analyses were consistent with the primary findings. After excluding 148 patients with malignancy, the association was attenuated but remained statistically significant (HR, 6.04, 95% CI, 2.42–15.08, Table S6). After applying OW, an eGFR dip ≥20% remained significantly associated with an increased risk of all-cause mortality within 45 days (HR, 7.41, 95% CI, 3.12–17.61). Finally, the *E*-value for the primary estimate was 15.64, indicating that an unmeasured confounder would need to be associated with both the exposure and all-cause mortality within 45 days by a risk ratio of at least 15.64 to fully explain away the observed association.

### Additional analyses

Furthermore, when alternative thresholds were applied, both eGFR decline ≥10% and ≥30% were associated with increased risks of all-cause mortality within 45 days (HR, 9.65; 95% CI, 3.24–28.72 and HR, 9.48; 95% CI, 4.30–20.90, respectively, Table S7).

## DISCUSSION

In this multicenter, real-world new-user study, we found that a pronounced short-term decline in eGFR following initiation of SGLT2i therapy was not uncommon, occurring in nearly one-quarter of patients. The incidence rates were higher in older individuals, patients with heart failure, those receiving diuretics, and patients with CKD stages 2–4. Importantly, an early eGFR dip ≥20% was independently associated with an eight-fold increased risk of all-cause mortality within 45 days, but not from 46 days to 1 year. When eGFR decline was analyzed as a continuous variable, we observed a linear association between the magnitude of eGFR decline and short-term mortality risk. Each 5% decrease in eGFR was associated with a 1.22-fold higher risk of all-cause mortality within 45 days, whereas no significant association was observed for mortality from 46 days to 1 year.

In this study, we found that an early decline in eGFR ≥20% following initiation of SGLT2i was associated with an increased risk of short-term (within 45 days) all-cause mortality, whereas no such association was observed among patients who survived beyond 45 days. Several explanations may account for this finding. First, it may reflect hemodynamic vulnerability in susceptible patients. SGLT2i reduce intraglomerular pressure through restoration of tubuloglomerular feedback, leading to an initial decline in eGFR that is generally considered hemodynamic and reversible [24]. However, in patients with impaired renal autoregulation, reduced effective circulating volume, or underlying cardiovascular instability (e.g. heart failure or concomitant diuretic use), this acute hemodynamic shift may be poorly tolerated [5]. In addition, short-term mortality risk may be mediated by AKI or rapid volume changes following SGLT2i initiation, which could further compromise renal perfusion and systemic hemodynamic stability [4, 5]. In such individuals, a pronounced decline in eGFR may indicate an inability to maintain adequate renal and circulatory homeostasis, thereby leading to early adverse outcomes in a subset of patients. Second, residual confounding may also contribute to the observed association. Patients with an eGFR decline ≥20% tended to have more fragile baseline characteristics, such as older age and a higher prevalence of heart failure. Although we applied IPTW-weighted Cox models to balance measured covariates, unmeasured or incompletely measured confounders may persist. Therefore, the increased mortality risk observed in this group may, at least in part, reflect underlying disease severity or clinical vulnerability rather than a direct causal effect of eGFR decline.

In real-world settings, the association between SGLT2i use and AKI remains controversial. Reports from the U.S. Food and Drug Administration (FDA) Adverse Event Reporting System (FAERS) suggested that SGLT2i use was associated with an increased risk of AKI compared with non-use, with nearly a three-fold higher reporting odds ratio (ROR, 2.88, 95% CI, 2.71–3.05) [25]. Among individual agents, canagliflozin was associated with a higher proportion of AKI reports (7.3%) compared with empagliflozin and dapagliflozin (4.7% and 4.8%, respectively; *P* < .001). In contrast, a 1:1 propensity score-matched (PSM) study comparing SGLT2i with other glucose-lowering drugs found no significant difference in incident AKI (defined as doubling of SCr or discharge diagnosis of AKI) between the two groups (HR, 0.64, 95% CI, 0.40–1.03) [26]. Nadkarni and colleagues further reported heterogeneous findings across two diabetic cohorts: in the Mount Sinai cohort, SGLT2i use was associated with a significantly lower risk of AKI compared with non-use (HR, 0.40, 95% CI, 0.20–0.70), whereas this association was attenuated and no longer statistically significant in the Geisinger cohort (HR, 0.60, 95% CI, 0.40–1.10) [27]. Moreover, a meta-analysis pooling four large empagliflozin RCTs demonstrated a 27% reduction in AKI adverse events compared with placebo (HR, 0.73, 95% CI, 0.63–0.85), with consistent effects across subgroups defined by diabetes status, baseline eGFR, UACR, and heart failure (all *P* for interaction >.05) [28]. Given the considerable heterogeneity across study populations, the lack of control groups in certain studies, and variability in AKI definitions, the association between SGLT2i use and subsequent AKI risk remains inconclusive.

Several observational studies have examined the prognostic implications of the early eGFR dip following SGLT2i initiation. A small single-center study from Japan (*n* = 21) reported that, among patients with type 2 diabetes mellitus and CKD with normoalbuminuria, an initial eGFR dip was independently associated with subsequent worsening of renal function in the SGLT2i group [29]. In addition, a meta-analysis including 8972 new SGLT2i users across six *post hoc* analyses of RCTs and one retrospective cohort study reported that 28%–41% of patients experienced an eGFR dip ≤10%, while 28%–49% experienced a dip >10% after treatment initiation [15]. Compared with patients without an eGFR decline, those with a dip ≤10% had a slightly lower risk of major adverse kidney events (HR, 0.92, 95% CI, 0.87–0.97), whereas those with a dip >10% had a similar risk (HR, 1.08, 95% CI, 0.72–1.61). Importantly, neither degree of eGFR dip was significantly associated with all-cause mortality in this study. However, our findings suggest that, compared with an eGFR dip <10%, even an eGFR dip >10% was associated with an increased risk of short-term mortality. These discrepancies may be attributable to differences in study populations, exposure classifications, and outcome definitions across studies. In a large Taiwanese observational study of 11 769 patients with type 2 diabetes mellitus initiating SGLT2i therapy, 62.9% experienced some degree of eGFR decline within 1–3 months, and 30.5%, 20.2%, 8.5%, and 3.7% of patients experienced an eGFR decline of 0%–10%, 10%–20%, 20%–30%, and >30%, respectively. [30]. Notably, only an eGFR decline >30% was associated with increased risks of new-onset atrial fibrillation, major adverse cardiovascular events/heart failure, and renal outcomes, whereas smaller declines (≤30%) were not associated with these outcomes. However, mortality data were unavailable in that study, and the present study provided additional evidence regarding this outcome.

Notably, most prior studies have focused on modest eGFR declines (typically using a 10% threshold), and whether larger eGFR dip confer distinct prognostic implications has remained unclear. In this multicenter real-world study, we found that ~22.5% of patients experienced an eGFR dip ≥20%, with the highest incidence observed among users of empagliflozin (25.1%), followed by dapagliflozin (22.7%) and canagliflozin (18.9%). Importantly, such a marked early decline was independently associated with an approximately eight-fold increased risk of short-term all-cause mortality.

This study leveraged multicenter real-world data to evaluate the association between early eGFR dip following SGLT2i initiation and all-cause mortality, with extensive subgroup analyses and multiple sensitivity analyses supporting the robustness of our findings. Our study has several limitations. First, as an observational study, residual confounding cannot be fully excluded. Despite the use of multivariable adjustment and weighting methods, residual confounding related to acute illness severity, including unmeasured hemodynamic instability, volume depletion, or intercurrent illness, cannot be completely ruled out. Unmeasured factors such as smoking, alcohol consumption, body mass index, use of nonsteroidal mineralocorticoid receptor antagonists, and glucagon-like peptide-1 receptor agonists might also influence the observed association between eGFR dip and mortality. However, the *E*-value of 15.64 suggested that unmeasured confounding of substantial magnitude would be required to fully explain away the observed association. Second, the median follow-up duration was relatively short (12.5 [5.7, 22.9] months), and the clinical significance of large early eGFR declines and longer-term mortality risk needs to further explore. Third, exposure to SGLT2i was inferred from prescription records, and kidney function was assessed using estimated rather than measured GFR, which may have introduced misclassification. Fourth, due to the study design, at least one SCr measurement within 14–28 days after initiation of SGLT2i was required, which may have introduced selection bias by excluding patients without follow-up testing, particularly those who were relatively healthier or had less intensive clinical monitoring. As a result, this could potentially lead to an overestimation of the association between early eGFR decline after SGLT2i initiation and short-term mortality in hospitalized patients with CKD. Fifth, due to the limited sample size and number of events in this study, the estimated CIs were relatively wide, and some subgroup analyses yielded non-estimable results. Therefore, the magnitude of the HR should be interpreted cautiously, even though the direction of association appeared robust across alternative definitions and sensitivity analyses. Larger studies are warranted to further validate our findings. Finally, although we observed an association between marked early eGFR decline and mortality, decisions regarding treatment continuation or discontinuation were beyond the scope of this study and warrant further investigation.

In conclusion, in this multicenter real-world study of patients with CKD stages 1–4, an early eGFR dip ≥20% after initiation of SGLT2i therapy was not uncommon and was independently associated with a higher short-term risk of all-cause mortality. These findings underscore the importance of close monitoring of kidney function after SGLT2i initiation. In patients who experience a substantial short-term decline in eGFR, careful clinical reassessment for potentially reversible causes, including hemodynamic instability, volume depletion, or intercurrent illness, should be considered rather than automatic discontinuation of therapy.

## Supplementary Material

sfag386_Supplemental_Files

## Data Availability

Please contact Honglan Wei at weihonglan2021@126.com or Junwu Dong at dongjunwu@126.com for access to the de-identified individual level data.
