# Supplementary material for: Substantial declines in eGFR after initiation of SGLT2-inhibitors associate with increased short-term mortality in hospitalized patients with CKD
Source: Clin Kidney J. 2026 Oct 29;19(11):sfag386. doi: 10.1093/ckj/sfag386 (PMC13768951; doi:10.1093/ckj/sfag386)
Supplement: Supplementary file 1 [file sfag386_supplemental_files.zip › Figure S1.pdf]

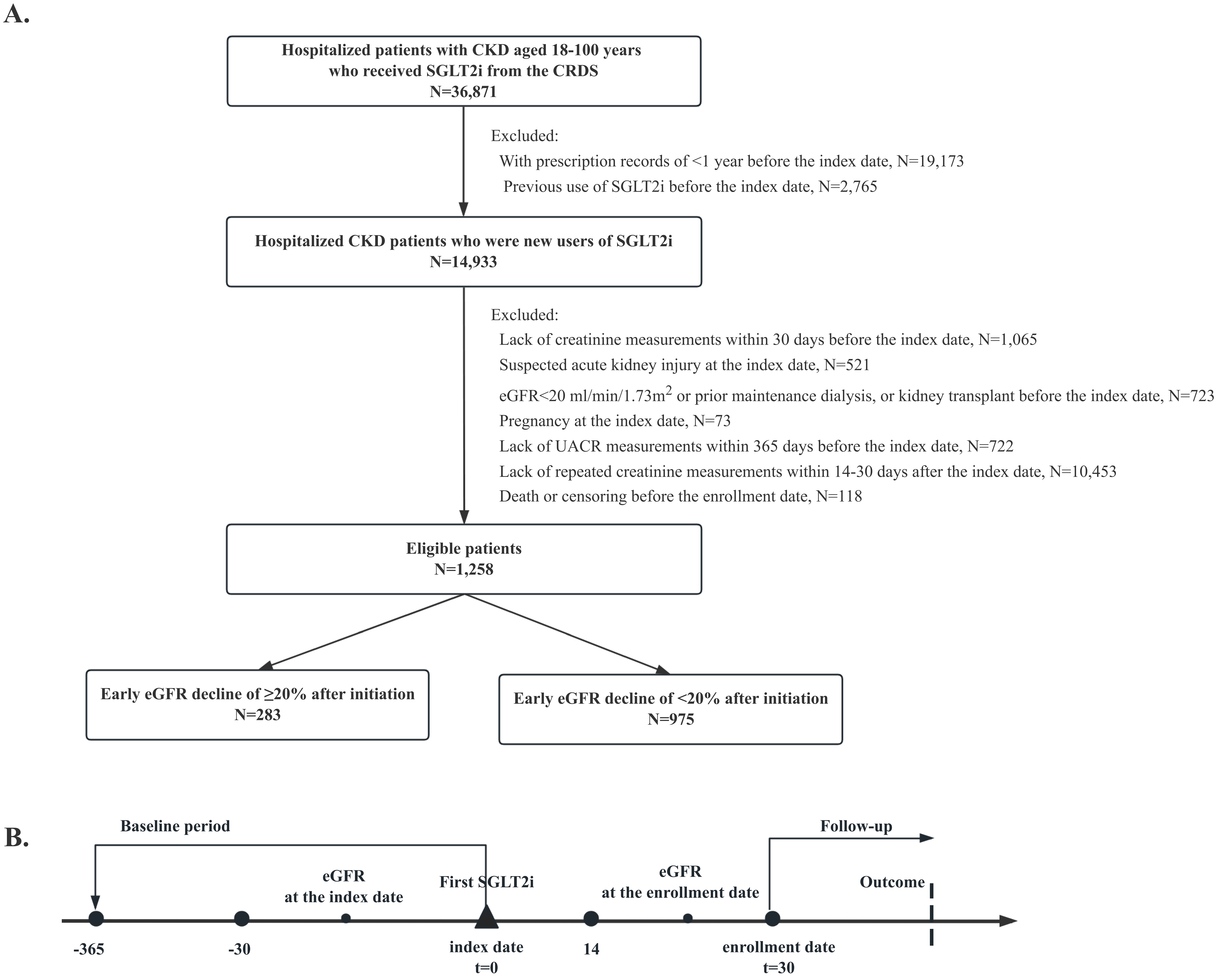
A.
Hospitalized patients with CKD aged 18-100 years
who received SGLT2i from the CRDS
N=36,871
Excluded:
With prescription records of <1 year before the index date, N=19,173
Previous use of SGLT2i before the index date, N=2,765
Hospitalized CKD patients who were new users of SGLT2i
N=14,933
Excluded:
Lack of creatinine measurements within 30 days before the index date, N=1,065
Suspected acute kidney injury at the index date, N=521
eGFR<20 ml/min/1.73m2 or prior maintenance dialysis, or kidney transplant before the index date, N=723
Pregnancy at the index date, N=73
Lack of UACR measurements within 365 days before the index date, N=722
Lack of repeated creatinine measurements within 14-30 days after the index date, N=10,453
Death or censoring before the enrollment date, N=118
Eligible patients
N=1,258
Early eGFR decline of ≥20% after initiation
N=283
Early eGFR decline of <20% after initiation
N=975
B.
Baseline period
Follow-up
eGFR
at the index date
First SGLT2i
eGFR
at the enrollment date
Outcome
-365
-30
index date
t=0
14
enrollment date
t=30
