# Supplementary material for: Substantial declines in eGFR after initiation of SGLT2-inhibitors associate with increased short-term mortality in hospitalized patients with CKD
Source: Clin Kidney J. 2026 Oct 29;19(11):sfag386. doi: 10.1093/ckj/sfag386 (PMC13768951; doi:10.1093/ckj/sfag386)
Supplement: Supplementary file 1 [file sfag386_supplemental_files.zip › Figure S2.pdf]

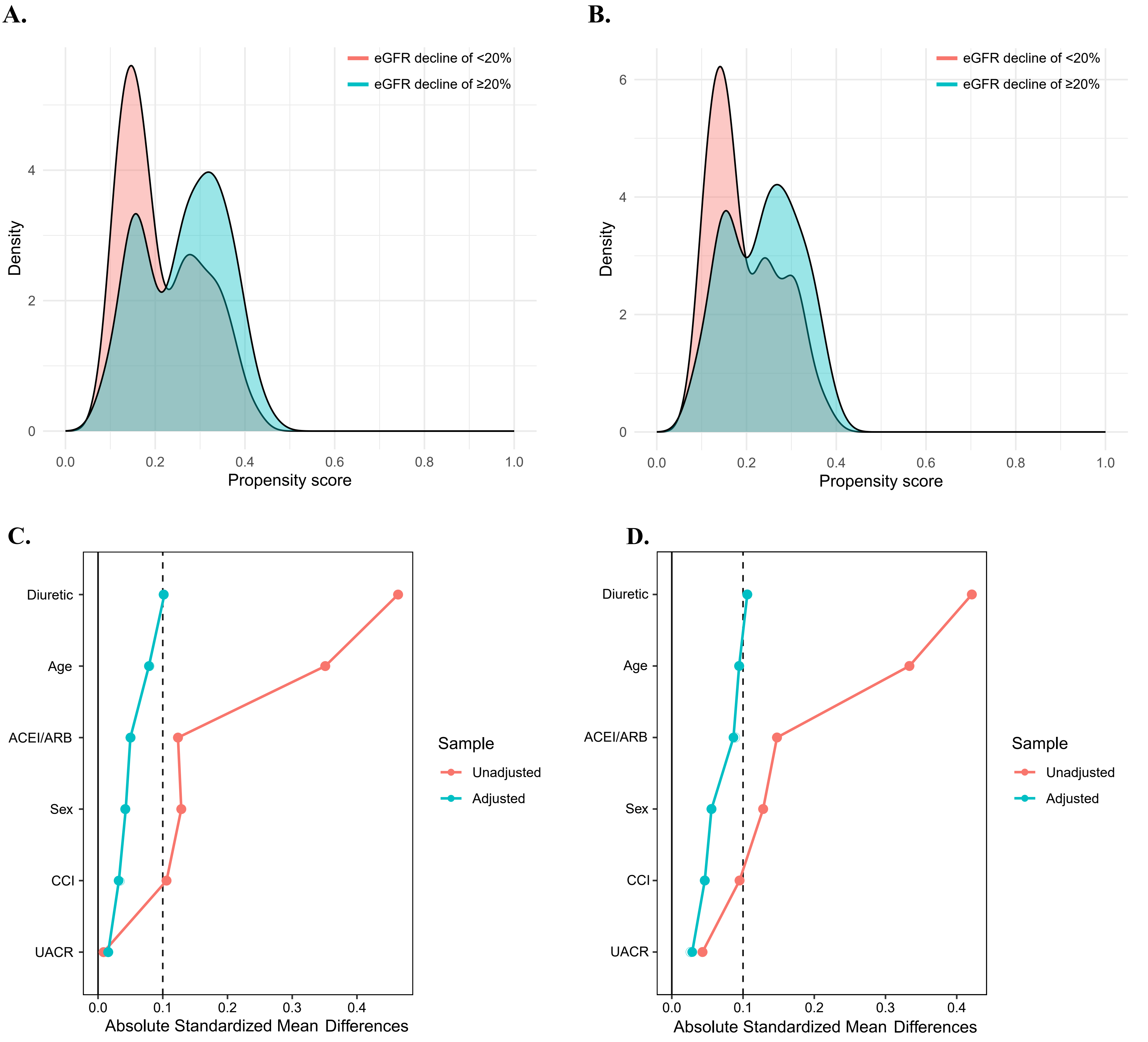

A.
B.
eGFR decline of <20%
eGFR decline of ≥20%
Density
Propensity score
0.0
0.2
0.4
0.6
0.8
1.0
0
2
4
6
C.
D.
Diuretic
Age
ACEI/ARB
Sex
CCI
UACR
Absolute Standardized Mean Differences
0.0
0.1
0.2
0.3
0.4
Sample
Unadjusted
Adjusted
