# Supplementary material for: Substantial declines in eGFR after initiation of SGLT2-inhibitors associate with increased short-term mortality in hospitalized patients with CKD
Source: Clin Kidney J. 2026 Oct 29;19(11):sfag386. doi: 10.1093/ckj/sfag386 (PMC13768951; doi:10.1093/ckj/sfag386)
Supplement: Supplementary file 1 [file sfag386_supplemental_files.zip › Supplemental Tables.docx]

**Table S1.** List of medications and diagnoses with corresponding ATC and ICD-10 codes

| Variable | ATC/ICD-10 codes ^*^ |
| --- | --- |
|  | **Medications (ATC codes)** |
| SGLT2i | A10BK |
| Dapagliflozin | A10BK01 |
| Canagliflozin | A10BK02 |
| Empagliflozin | A10BK03 |
| Ertugliflozin | A10BK04 |
| ACEI/ARB | C09 |
| Beta block | C07 |
| Calcium channel blocker | C08 |
| Diuretic | C03 |
| Insulin | A10A |
| Metformin | A10BA02 |
| DPP4i | A10BH |
| Sulfonylurea | A10BB |
| Statin | C10AA |
| NSAIDs | M01A\|A07EC\|N02B |
| Proton pump inhibitor | A02BC |
| Contrast | V08 |
|  | **Diagnoses (ICD-10 codes)** |
| Diabetes mellitus | E10-E14 |
| Hypertension | H35.004\|I10-I15\|I61.902\|I67.4\|P29.2\|T70.202 |
| Heart failure | I11.0\|I13.0\|I13.2\|I50\|I97.102\|I97.803\|P29.0\|R57.0 |
| Acute coronary syndrome | I20.0\|I21-I25 |
| Cerebrovascular disease | D18.001\|D18.002\|F06.910\|G45\|G46\|I60-I69\|I77.302\|Q28.0-Q28.3\|Z92.401 |
| Stroke | I60\|I61\|I62\|I63\|I64\|I69 |
| COPD | J44 |
| Malignant tumor | C00-C97\|D00-D48\|K76.814\|Z86.0\|Z92.402\|Z92.6 |

**Abbreviations**: ATC, anatomical therapeutic chemical classification system; ICD-10, international classification of diseases, 10th revision; SGLT2i, sodium-glucose cotransporter 2 inhibitors; COPD, chronic obstructive pulmonary disease; ACEI/ARB, angiotensin-converting enzyme inhibitor/angiotensin receptor blocker; DPP4i, dipeptidyl peptidase 4 inhibitors; NSAIDs, nonsteroidal anti-inflammatory drug.

**Table S2. Missingness of baseline covariates**

| Variable | Count (rate, %) ^*^ |
| --- | --- |
| SGLT2i subtypes | 0 (0) |
| Age | 0 (0) |
| SCr at index date | 0 (0) |
| eGFR at index date | 0 (0) |
| CKD stages | 0 (0) |
| eGFR at enrollment date | 0 (0) |
| UACR | 0 (0) |
| Charlson comorbidity index | 0 (0) |
| Diabetes mellitus | 0 (0) |
| Hypertension | 0 (0) |
| Heart failure | 0 (0) |
| Acute coronary syndrome | 0 (0) |
| Cerebrovascular disease | 0 (0) |
| Stroke | 0 (0) |
| COPD | 0 (0) |
| Malignant tumor | 0 (0) |
| ACEI/ARB | 0 (0) |
| Beta block | 0 (0) |
| Calcium channel blocker | 0 (0) |
| Diuretic | 0 (0) |
| Insulin | 0 (0) |
| Metformin | 0 (0) |
| DPP4i | 0 (0) |
| Sulfonylurea | 0 (0) |
| Statin | 0 (0) |
| NSAIDs | 0 (0) |
| Proton pump inhibitor | 0 (0) |
| Contrast | 0 (0) |
| Cardiac major surgery | 0 (0) |
| CABG | 0 (0) |
| BUN | 1 (0.1) |
| Hemoglobin | 2 (0.2) |
| Potassium | 11 (0.9) |
| Uric acid | 11 (0.9) |
| Sodium | 13 (1.0) |
| Albumin | 15 (1.2) |
| Glucose | 26 (2.1) |
| Total cholesterol | 44 (3.5) |
| Triglycerides | 61 (4.8) |
| LDL-C | 75 (6.0) |
| HDL-C | 76 (6.0) |
| SBP | 84 (6.7) |
| DBP | 87 (6.9) |
| D-dimer | 215 (17.1) |

^*^Missing proportion was calculated as the number of missing observations for each variable divided by the total study population (N = 1,258).

**Abbreviations**: CKD, chronic kidney disease; eGFR, estimated glomerular filtration rate; SCr, serum creatinine; UACR, urine albumin-to-creatinine ratio; SGLT2i, sodium-glucose cotransporter 2 inhibitors; SBP, systolic blood pressure; DBP, diastolic blood pressure; COPD, chronic obstructive pulmonary disease; ACEI/ARB, angiotensin-converting enzyme inhibitor/angiotensin receptor blocker; DPP4i, dipeptidyl peptidase 4 inhibitors; NSAIDs, nonsteroidal anti-inflammatory drugs; CABG, coronary artery bypass grafting; BUN, blood urea nitrogen; LDL-C, low density lipoprotein cholesterol; HDL-C, high density lipoprotein cholesterol.

**Table S3.** Baseline characteristics of patients with CKD stratified by all-cause mortality

| Variable | All-cause mortality within 45 days | | All-cause mortality from 46 days to 1 year | |
| --- | --- | --- | --- | --- |
|  | No (n=1,230) | Yes (n=28) | No (n=1,090) | Yes (n=60) |
| Age, year | 64.3 (14.5) | 71.8 (11.8) | 63.6 (14.3) | 74.5 (13.8) |
| Male, n (%) | 856 (69.6) | 16 (57.1) | 758 (69.5) | 50 (83.3) |
| SCr at index date, mmol/L | 99.0 [76.0, 127.0] | 98.5 [71.1, 120.] | 99.0 [76.0, 126.4] | 93.7 [70.8, 141.2] |
| eGFR at index date, mL/min/1.73m^2^ | 66.9 [49.7, 89.9] | 58.4 [52.7, 91.2] | 66.9 [50.3, 89.8] | 67.2 [40.6, 90.8] |
| CKD stages, n (%) |  |  |  |  |
| 1 | 305 (24.8) | 8 (28.6) | 268 (24.6) | 17 (28.3) |
| 2 | 425 (34.6) | 5 (17.9) | 386 (35.4) | 15 (25.0) |
| 3 | 432 (35.1) | 14 (50.0) | 378 (34.7) | 22 (36.7) |
| 4 | 68 (5.5) | 1 (3.6) | 58 (5.3) | 6 (10.0) |
| eGFR at enrollment date, mL/min/1.73m^2^ | 63.7 [44.1, 86.5] | 42.4 [26.1, 64.9] | 64.0 [45.0, 86.6] | 58.7 [36.2, 83.7] |
| UACR, mg/g | 75.5 [15.3, 602.8] | 68.5 [15.0, 293.4] | 75.5 [15.3, 627.9] | 72.5 [14.1, 368.1] |
| SGLT2i subtypes, n (%) |  |  |  |  |
| Dapagliflozin | 821 (66.7) | 20 (71.4) | 721 (66.1) | 41 (68.3) |
| Canagliflozin | 173 (14.1) | 2 (7.1) | 158 (14.5) | 9 (15.0) |
| Empagliflozin | 229 (18.6) | 6 (21.4) | 205 (18.8) | 10 (16.7) |
| Ertugliflozin | 7 (0.6) | 0 (0.0) | 6 (0.6) | 0 (0.0) |
| SBP, mmHg | 131.7 (21.2) | 119.6 (25.0) | 131.8 (21.1) | 128.4 (21.3) |
| DBP, mmHg | 77.2 (13.4) | 71.5 (11.4) | 77.5 (13.4) | 70.6 (11.9) |
| Charlson comorbidity index | 8.0 [5.0, 10.0] | 9.5 [7.0, 12.0] | 8.0 [5.0, 10.0] | 10.0 [7.0, 13.0] |
| Diabetes mellitus, n (%) | 911 (74.1) | 18 (64.3) | 816 (74.9) | 47 (78.3) |
| Hypertension, n (%) | 809 (65.8) | 20 (71.4) | 706 (64.8) | 46 (76.7) |
| Heart failure, n (%) | 387 (31.5) | 19 (67.9) | 322 (29.5) | 34 (56.7) |
| Acute coronary syndrome, n (%) | 490 (39.8) | 17 (60.7) | 431 (39.5) | 34 (56.7) |
| Cerebrovascular disease, n (%) | 429 (34.9) | 10 (35.7) | 370 (33.9) | 27 (45.0) |
| Stroke, n (%) | 320 (26.0) | 10 (35.7) | 267 (24.5) | 27 (45.0) |
| COPD, n (%) | 251 (20.4) | 7 (25.0) | 218 (20.0) | 18 (30.0) |
| Malignant tumor, n (%) | 144 (11.7) | 4 (14.3) | 122 (11.2) | 15 (25.0) |
| ACEI/ARB, n (%) | 837 (68.0) | 19 (67.9) | 746 (68.4) | 39 (65.0) |
| Beta block, n (%) | 536 (43.6) | 18 (64.3) | 461 (42.3) | 35 (58.3) |
| Calcium channel blocker, n (%) | 639 (52.0) | 16 (57.1) | 563 (51.7) | 33 (55.0) |
| Diuretic, n (%) | 606 (49.3) | 20 (71.4) | 510 (46.8) | 46 (76.7) |
| Insulin, n (%) | 729 (59.3) | 17 (60.7) | 644 (59.1) | 41 (68.3) |
| Metformin, n (%) | 436 (35.4) | 7 (25.0) | 395 (36.2) | 17 (28.3) |
| DPP4i, n (%) | 253 (20.6) | 3 (10.7) | 222 (20.4) | 21 (35.0) |
| Sulfonylurea, n (%) | 179 (14.6) | 2 (7.1) | 161 (14.8) | 11 (18.3) |
| Statin, n (%) | 876 (71.2) | 19 (67.9) | 772 (70.8) | 48 (80.0) |
| NSAIDs, n (%) | 642 (52.2) | 17 (60.7) | 568 (52.1) | 35 (58.3) |
| Proton pump inhibitor, n (%) | 589 (47.9) | 21 (75.0) | 501 (46.0) | 44 (73.3) |
| Contrast, n (%) | 277 (22.5) | 8 (28.6) | 242 (22.2) | 11 (18.3) |
| Cardiac major surgery, n (%) | 11 (0.9) | 2 (7.1) | 10 (0.9) | 0 (0.0) |
| CABG, n (%) | 56 (4.6) | 4 (14.3) | 37 (3.4) | 11 (18.3) |
| BUN, mmol/L | 7.2 [5.5, 9.5] | 7.7 [5.9, 11.1] | 7.1 [5.5, 9.4] | 7.8 [6.0, 11.0] |
| Uric acid, mmol/L | 380.0 [301.0, 475.0] | 363.0 [257.8, 473.5] | 380.5 [301.0, 473.0] | 390.5 [308.0, 501.8] |
| Glucose, mmol/L | 7.7 [5.7, 11.3] | 8.7 [5.3, 11.8] | 7.7 [5.8, 11.4] | 9.0 [6.4, 11.8] |
| Triglycerides, mmol/L | 1.5 [1.1, 2.4] | 1.2 [0.9, 1.9] | 1.6 [1.1, 2.5] | 1.3 [1.0, 1.8] |
| Total cholesterol, mmol/L | 4.4 [3.5, 5.5] | 3.9 [2.7, 4.9] | 4.4 [3.5, 5.5] | 4.0 [3.4, 4.7] |
| LDL-C, mmol/L | 2.6 [1.9, 3.3] | 2.1 [1.4, 3.0] | 2.6 [1.9, 3.4] | 2.3 [1.8, 3.0] |
| HDL-C, mmol/L | 1.0 [0.9, 1.3] | 0.9 [0.8, 1.3] | 1.0 [0.9, 1.2] | 1.0 [0.8, 1.3] |
| Albumin, g/L | 38.1 [33.6, 41.4] | 32.7 [29.0, 37.5] | 38.3 [33.8, 41.7] | 35.6 [31.5, 38.6] |
| Hemoglobin, g/L | 128.0 [112.0, 143.8] | 111.5 [89.8, 133.0] | 129.0 [114.0, 144.0] | 116.5 [99.0, 131.5] |
| Sodium, mmol/L | 139.6 [137.1, 141.7] | 138.3 [135.0, 141.2] | 139.8 [137.3, 141.7] | 139.0 [136.3, 140.7] |
| Potassium, mmol/L | 4.1 [3.7, 4.4] | 3.9 [3.6, 4.3] | 4.1 [3.7, 4.3] | 4.0 [3.7, 4.4] |
| D-dimer, μg/mL | 0.5 [0.3, 1.1] | 1.2 [0.7, 2.0] | 0.5 [0.3, 1.0] | 1.1 [0.5, 2.2] |

Data were presented as mean (standard deviation), median [25^th^, 75^th^ percentiles], or frequencies (percentages).

**Abbreviations**: CKD, chronic kidney disease; eGFR, estimated glomerular filtration rate; SCr, serum creatinine; UACR, urine albumin-to-creatinine ratio; SGLT2i, sodium-glucose cotransporter 2 inhibitors; SBP, systolic blood pressure; DBP, diastolic blood pressure; COPD, chronic obstructive pulmonary disease; ACEI/ARB, angiotensin-converting enzyme inhibitor/angiotensin receptor blocker; DPP4i, dipeptidyl peptidase 4 inhibitors; NSAIDs, nonsteroidal anti-inflammatory drugs; CABG, coronary artery bypass grafting; BUN, blood urea nitrogen; LDL-C, low density lipoprotein cholesterol; HDL-C, high density lipoprotein cholesterol.

**Table S4.** Schoenfeld residuals tests for the association between early eGFR decline and all-cause mortality in Model 2 (IPTW-weighted landmark Cox model with 45-day piecewise analysis).

|  | Chisq statistics ^*^ | P value | Global Chisq statistics ^*^ | Global P value |
| --- | --- | --- | --- | --- |
| **All-cause mortality within 45 days** | | | | |
| <20% vs. ≥20% | 0.041 | 0.840 | 6.705 | 0.460 |
| **All-cause mortality from 46 days to 1 year** | | | | |
| <20% vs. ≥20% | 0.077 | 0.782 | 8.810 | 0.267 |

^*^ Chisq statistic for the exposure variable; Global Chisq statistics for the entire model.

**Abbreviations**: HR, hazard ratio; CI, confidence interval; Ref, reference; IPTW, inverse probability of treatment weighting; CCI, Charlson comorbidity index; UACR, urine albumin-to-creatinine ratio; ACEI/ARB, angiotensin-converting enzyme inhibitor/angiotensin receptor blocker.

**Model 2**: adjusted for sex, age, CCI, UACR, ACEI/ARB, and diuretic.

**Table S5. Subgroup analyses:** Association between early eGFR decline and risk of all-cause mortality using a 45-day piecewise analysis with IPTW-weighted landmark Cox models in hospitalized patients with CKD

| Subgroups | No. of Events  (per 100 person-months) | | HR [95% CI] ^#^ | P for interaction |
| --- | --- | --- | --- | --- |
|  | Initial dip in eGFR | |  |  |
|  | ≥20% | <20% |  |  |
| **All-cause mortality within 45 days** | | | | |
| Age, years |  |  |  | 0.536 |
| ≤60 (n=461) | 3 (2.8) | 1 (0.2) | 14.39 [1.49, 139.10] |  |
| >60 (n=797) | 17 (6.2) | 7 (0.8) | 6.87 [2.80, 16.88] |  |
| Sex |  |  |  | 0.091 |
| Female (n=386) | 7 (5.1) | 5 (1.2) | 3.58 [1.12, 11.49] |  |
| Male (n=872) | 13 (5.3) | 3 (0.3) | 15.13 [4.26, 53.82] |  |
| CKD stages |  |  |  | 0.133 |
| 1-2 (n=743) | 7 (3.5) | 6 (0.7) | 4.32 [1.43, 13.05] |  |
| 3-4 (n=515) | 13 (7.2) | 2 (0.4) | 16.57 [3.69, 74.34] |  |
| UACR, mg/g |  |  |  | 0.263 |
| <300 (n=821) | 14 (5.5) | 7 (0.8) | 5.96 [2.35, 15.11] |  |
| ≥300 (n=437) | 6 (4.7) | 1 (0.2) | 19.94 [2.39, 166.51] |  |
| SGLT2i subtypes | |  |  | 0.741 |
| Dapagliflozin (n=841) | 14 (5.5) | 6 (0.6) | 7.06 [2.66, 18.73] |  |
| Non-dapagliflozin (n=417) | 6 (4.8) | 2 (0.4) | 9.64 [1.92, 48.33] |  |
| Hypertension |  |  |  | 0.651 |
| No (n=429) | 6 (4.7) | 2 (0.4) | 10.45 [2.06, 52.90] |  |
| Yes (n=829) | 14 (5.5) | 6 (0.6) | 6.83 [2.58, 18.07] |  |
| Diabetes mellitus |  |  |  | 0.717 |
| No (n=329) | 8 (7.2) | 2 (0.6) | 9.52 [1.97, 45.94] |  |
| Yes (n=929) | 12 (4.4) | 6 (0.6) | 6.81 [2.52, 18.41] |  |
| Heart failure |  |  |  | 0.138 |
| No (n=852) | 7 (3.4) | 2 (0.2) | 17.00 [3.51, 82.45] |  |
| Yes (n=406) | 13 (7.3) | 6 (1.5) | 4.43 [1.65, 11.92] |  |
| ACEI/ARB |  |  |  | 0.355 |
| No (n=402) | 7 (6.8) | 2 (0.4) | 13.83 [2.81, 68.10] |  |
| Yes (n=856) | 13 (4.6) | 6 (0.6) | 5.91 [2.22, 15.73] |  |
| Diuretic |  |  |  | 0.633 |
| No (n=632) | 4 (3.1) | 4 (0.5) | 5.87 [1.46, 23.59] |  |
| Yes (n=626) | 16 (6.3) | 4 (0.6) | 9.02 [3.00, 27.09] |  |
| **All-cause mortality from 46 days to 1 year** | | | | |
| Age, years |  |  |  | NA |
| ≤60 (n=431) | 0 (0.0) | 7 (0.2) | NA |  |
| >60 (n=719) | 14 (1.0) | 39 (0.9) | 1.01 [0.55, 1.88] |  |
| Sex |  |  |  | 0.197 |
| Female (n=342) | 4 (0.6) | 6 (0.3) | 2.01 [0.56, 7.16] |  |
| Male (n=808) | 10 (0.8) | 40 (0.7) | 0.76 [0.37, 1.53] |  |
| CKD stages |  |  |  | 0.596 |
| 1-2 (n=686) | 6 (0.6) | 26 (0.6) | 0.78 [0.32, 1.91] |  |
| 3-4 (n=464) | 8 (0.9) | 20 (0.7) | 1.08 [0.47, 2.46] |  |
| UACR, mg/g |  |  |  | 0.547 |
| <300 (n=745) | 9 (0.7) | 32 (0.7) | 0.81 [0.38, 1.71] |  |
| ≥300 (n=405) | 5 (0.8) | 14 (0.5) | 1.20 [0.43, 3.36] |  |
| SGLT2i subtypes |  |  |  | 0.159 |
| Dapagliflozin (n=762) | 12 (1.0) | 29 (0.6) | 1.18 [0.59, 2.32] |  |
| Non-dapagliflozin (n=388) | 2 (0.3) | 17 (0.6) | 0.40 [0.09, 1.76] |  |
| Hypertension |  |  |  | 0.031 |
| No (n=429) | 6 (1.0) | 8 (0.3) | 2.66 [0.90, 7.83] |  |
| Yes (n=829) | 8 (0.6) | 38 (0.8) | 0.61 [0.28, 1.31] |  |
| Diabetes mellitus |  |  |  | 0.533 |
| No (n=329) | 4 (0.9) | 9 (0.5) | 1.29 [0.39, 4.21] |  |
| Yes (n=929) | 10 (0.7) | 37 (0.6) | 0.83 [0.41, 1.68] |  |
| Heart failure |  |  |  | 0.796 |
| No (n=852) | 5 (0.5) | 21 (0.4) | 0.97 [0.36, 2.59] |  |
| Yes (n=406) | 9 (1.1) | 25 (1.2) | 0.82 [0.38, 1.77] |  |
| ACEI/ARB |  |  |  | 0.998 |
| No (n=365) | 5 (1.0) | 16 (0.7) | 0.92 [0.33, 2.58] |  |
| Yes (n=785) | 9 (0.7) | 30 (0.6) | 0.92 [0.44, 1.95] |  |
| Diuretic |  |  |  | 0.467 |
| No (n=594) | 1 (0.1) | 13 (0.3) | 0.49 [0.06, 3.71] |  |
| Yes (n=556) | 13 (1.1) | 33 (1.0) | 1.01 [0.53, 1.92] |  |

^*^A 45-day piecewise analysis was applied, with follow-up divided into 0-45 days and 46 days to 1 year.

**Abbreviations**: eGFR, estimated glomerular filtration rate; IPTW, inverse probability of treatment weighting; CKD, chronic kidney disease; HR, hazard ratio; CI, confidence interval; Ref, reference; CCI, Charlson comorbidity index; UACR, urine albumin-to-creatinine ratio; ACEI/ARB, angiotensin-converting enzyme inhibitor/angiotensin receptor blocker.

**^*^** Adjusted for sex, age, CCI, UACR, ACEI/ARB, and diuretic.

**Table S6. Sensitivity analyses:** Association between early eGFR decline and risk of all-cause mortality using a 45-day piecewise analysis in hospitalized patients with CKD

| Initial dip in eGFR | Total, N | No. of Events  (per 100 person-months) | **Crude model** | | **Model 1** | | **Model 2** | |
| --- | --- | --- | --- | --- | --- | --- | --- | --- |
|  |  |  | HR [95% CI] | P value | HR [95% CI] | P value | HR [95% CI] | P value |
| **Sensitivity analysis 1: excluding patients with malignant tumor (n=1,110) ^#^** | | | | | | | | |
| **All-cause mortality within 45 days** | | | | | | | | |
| <20% | 851 | 8 (0.7) | Ref |  | Ref |  | Ref |  |
| ≥20% | 259 | 16 (4.6) | 6.59 [2.76, 15.77] | <0.001 | 6.29 [2.58, 15.35] | <0.001 | 6.04 [2.42, 15.08] | <0.001 |
| **All-cause mortality from 46 days to 1 year** | | | | | | | | |
| <20% | 797 | 34 (0.5) | Ref |  | Ref |  | Ref |  |
| ≥20% | 216 | 11 (0.6) | 1.04 [0.52, 2.08] | 0.904 | 0.91 [0.45, 1.82] | 0.785 | 0.76 [0.39, 1.49] | 0.423 |
| **Sensitivity analysis 2: using overlap weighting (n=1,257)** | | | | | | | | |
| **All-cause mortality within 45 days** | | | | | | | | |
| <20% | 975 | 8 (0.6) | Ref |  | Ref |  | Ref |  |
| ≥20% | 283 | 20 (5.2) | 7.30 [3.07, 17.36] | <0.001 | 7.40 [3.12, 17.56] | <0.001 | 7.41 [3.12, 17.61] | <0.001 |
| **All-cause mortality from 46 days to 1 year** | | | | | | | | |
| <20% | 915 | 46 (0.6) | Ref |  | Ref |  | Ref |  |
| ≥20% | 235 | 14 (0.8) | 0.97 [0.53, 1.77] | 0.909 | 0.99 [0.54, 1.82] | 0.985 | 0.94 [0.51, 1.72] | 0.839 |

**^#^**Sensitivity analyses 1 was conducted using IPTW-weighted landmark Cox proportional hazards models.

**Abbreviations**: eGFR, estimated glomerular filtration rate; CKD, chronic kidney disease; HR, hazard ratio; CI, confidence interval; Ref, reference; IPTW, inverse probability of treatment weighting; CCI, Charlson comorbidity index; UACR, urine albumin-to-creatinine ratio; ACEI/ARB, angiotensin-converting enzyme inhibitor/angiotensin receptor blocker; EPV, events per variable.

**Model 1**: adjusted for sex, age, and CCI.

**Model 2**: Model 1+further adjusted for UACR, ACEI/ARB, and diuretic.

For sensitivity analysis 1, the EPV values in Model 2 were 3.4 for all-cause mortality within 45 days and 6.4 for all-cause mortality from 46 days to 1 year. For sensitivity analysis 2, the corresponding EPV values in Model 2 were 4 and 8.6, respectively.

**Table S7. Additional analyses:** Association between early eGFR decline and risk of all-cause mortality using alternative definitions of eGFR decline in a 45-day piecewise analysis among hospitalized patients with CKD

| Initial dip in eGFR | Total, N | No. of Events  (per 100 person-months) | **Crude model** | | **Model 1** | | **Model 2** | |
| --- | --- | --- | --- | --- | --- | --- | --- | --- |
|  |  |  | HR [95% CI] | P value | HR [95% CI] | P value | HR [95% CI] | P value |
| **Additional analysis 1: Using a ≥10% decline in eGFR as the exposure definition (n = 1,257) ^#^** | | | | | | | | |
| **All-cause mortality within 45 days** | | | | | | | | |
| <10% | 775 | 4 (0.4) | Ref |  | Ref |  | Ref |  |
| ≥10% | 483 | 24 (3.6) | 10.85 [3.72, 31.66] | <0.001 | 9.99 [3.39, 29.46] | <0.001 | 9.65 [3.24, 28.72] | <0.001 |
| **All-cause mortality from 46 days to 1 year** | | | | | | | | |
| <10% | 728 | 36 (0.6) | Ref |  | Ref |  | Ref |  |
| ≥10% | 422 | 24 (0.7) | 1.06 [0.63, 1.80] | 0.814 | 0.91 [0.54, 1.53] | 0.723 | 0.84 [0.50, 1.41] | 0.512 |
| **Additional analysis 2: Using a ≥30% decline in eGFR as the exposure definition (n = 1,257) ^#^** | | | | | | | | |
| **All-cause mortality within 45 days** | | | | | | | | |
| <30% | 1,099 | 12 (0.8) | Ref |  | Ref |  | Ref |  |
| ≥30% | 159 | 16 (7.7) | 9.93 [4.61, 21.39] | <0.001 | 9.60 [4.36, 21.14] | <0.001 | 9.48 [4.30, 20.90] | <0.001 |
| **All-cause mortality from 46 days to 1 year** | | | | | | | | |
| <30% | 1,024 | 52 (0.6) | Ref |  | Ref |  | Ref |  |
| ≥30% | 126 | 8 (0.9) | 1.09 [0.50, 2.35] | 0.827 | 1.00 [0.45, 2.19] | 0.993 | 0.89 [0.42, 1.88] | 0.752 |

**^#^**Additional analyses 1 and 2 were conducted using IPTW-weighted landmark Cox proportional hazards models.

**Abbreviations**: eGFR, estimated glomerular filtration rate; CKD, chronic kidney disease; HR, hazard ratio; CI, confidence interval; Ref, reference; IPTW, inverse probability of treatment weighting; CCI, Charlson comorbidity index; UACR, urine albumin-to-creatinine ratio; ACEI/ARB, angiotensin-converting enzyme inhibitor/angiotensin receptor blocker; EPV, events per variable.

**Model 1**: adjusted for sex, age; and CCI.

**Model 2**: Model 1+further adjusted for UACR, ACEI/ARB, and diuretic.

For additional analyses 1, 2, and 3, the EPV values for Model 2 were 4 for all-cause mortality within 45 days and 8.6 for all-cause mortality from 46 days to 1 year.
